# Asthma and subsequent school performance at age 15–16 years: A Swedish population-based sibling control study

**DOI:** 10.1038/s41598-020-64633-w

**Published:** 2020-05-06

**Authors:** Cecilia Lundholm, Bronwyn K. Brew, Brian M. D’Onofrio, Emma Caffrey Osvald, Henrik Larsson, Catarina Almqvist

**Affiliations:** 1 Department of Medical Epidemiology and Biostatistics, Karolinska Institutet, Stockholm, Sweden; 2 National Perinatal Epidemiology and Biostatistics Unit, Centre for Big Data Research in Health and School of Women and Children’s Health, University of New South Wales, Sydney, Australia; 3 Department of Psychological and Brain Sciences, Indiana University, Bloomington, IN USA; 4 Pediatric Allergy and Pulmonology Unit at Astrid Lindgren Children’s Hospital, Karolinska University Hospital, Stockholm, Sweden; 5 School of Medical Sciences, Örebro University, Örebro, Sweden

**Keywords:** Medical research, Risk factors

## Abstract

Asthma may negatively affect children’s school performance, such as grades and exam results. Results from previous studies have shown varying results and may have suffered from confounding and other biases. We used a Swedish population-based cohort of 570,595 children with data on asthma (including severity and control) in Grades 7–8 and 9, school performance from Grade 9 (grade point sum, non-eligibility for upper secondary school and national test results) and measured confounders from national registers. We used sibling comparisons to account for unmeasured familial factors. Children with asthma and severe asthma performed slightly better in school than children without asthma when adjusting for measured confounders, but the associations were attenuated in sibling comparisons. In contrast, children with uncontrolled asthma performed slightly worse (e.g. Grade 9: β_adj_ = −9.9; 95% CI −12.8 to −7.0; Cohen’s d = 0.16). This association remained for uncontrolled asthma in Grade 9 in sibling comparisons (Grade 9: β = −7.7 points; 95% CI −12.6 to −2.6; Cohen’s d = 0.12), but not for Grades 7–8. The attenuation of estimates when controlling for familial factors using sibling comparisons suggests that the differences were due to familial factors, rather than being causal. The remaining associations in sibling comparisons between uncontrolled asthma in Grade 9 and school performance are consistent with a causal association.

## Introduction

Asthma is the most common chronic illness in childhood. Symptoms include shortness of breath, chest tightness, cough^1^ and sleep disturbance due to breathing difficulties^2^, which could affect school performance, such as grades and exam results.

Studies have shown that children with asthma have a higher degree of absenteeism from school^3–9^. Children with persistent^5^ or uncontrolled^7^ asthma are more absent from school, as well as children with more severe asthma^4^, compared to children with milder controlled asthma. Children with severe or uncontrolled asthma are also more likely to have allergic asthma^10^, with increased symptoms during the pollen season in the spring, when many important exams take place. Both asthma symptoms and school absenteeism may have a detrimental effect on school results. Further, living with a chronic illness, such as asthma, can also create stress, which may influence the child’s school performance^11^.

Studies on the association between asthma and school performance have shown varying results. Two reviews concluded that there was no or a very small association between asthma and school performance^3,4^. However, a recent meta-analysis in children with asthma showed lower cognitive function in several domains compared to their healthy peers, including global intellect, academic achievement and executive functioning^12^. Although studies in the last few years have also provided evidence of worse school performance in children with asthma compared to children without^6,9,13^, a study using Swedish twins found no evidence of association between asthma and school performance^14^. Not only is it unclear whether asthma effects academic performance, there are a number of other related knowledge gaps. The effect of severe or uncontrolled asthma has been difficult to assess due to few affected children^13,14^. Secondly, it is well-established that attention deficit hyperactivity disorder (ADHD) is more common in children with asthma^15–18^ and that children with ADHD perform worse in school than children without ADHD^19,20^. Irani *et al*. concluded there is a lack of studies taking asthma control and comorbidities, such as ADHD, into account^12^. Finally, it is also unclear whether children with asthma fall behind in school such that asthma in lower grades has a lasting effect on school results several years later. Most previous studies have been cross-sectional and included children in wide age ranges, which also may introduce cohort-effects.

Furthermore, many studies have been unable to account for other important confounders. It is known that children of parents with lower socio-economic status achieve lower school performance^21^. There is also evidence of an association between parental socio-economic status and asthma^22,23^. This opens up for potential confounding by socio-economic status and other family factors in the association between asthma and school performance. Family (familial) factors such as parent characteristics, home environment and family life style are often difficult to measure accurately; therefore, we need more advanced methods than ordinary adjustment in regression models to account for them. Fortunately, those factors are, to a large extent shared by siblings. Thus, we can use sibling comparison to control for such unmeasured familial factors^24^. Sibling analysis has been used in a small study from the United States, with no difference in school results between the children with asthma and their siblings without asthma^25^, suggesting that the association is not causal.

In Sweden, registers with information on school results, health issues and family relations exist for the entire population. The registers can be linked using the Swedish personal identity number, unique for each Swedish resident^26^, making it possible to perform large-scale epidemiological total population studies.

We hypothesised that children with asthma have lower school performance than their peers without asthma. We therefore aimed to estimate the association between asthma, overall and sub-divided by severity and control, during two different time intervals (school Grades 7–8 and 9) and measures of school performance in Grade 9, such as grade point sum, eligibility to upper secondary school and results of national tests in the three core subjects (English, mathematics and Swedish). We combined measured covariates, e.g. ADHD and parental socio-economy, and a sibling comparison approach to account for familial confounding. Further, we aimed to explore potential differences in the associations between asthma and school performance by parental socio-economic group and ADHD in the child.

## Methods

### Study population

**Figure 1:**
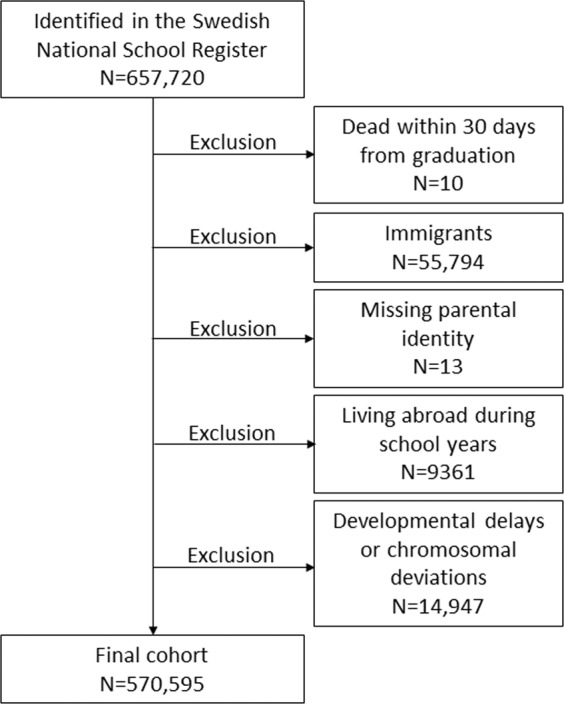
Flow chart of study population.

This population-based register study included all children with grades in Grade 9 of compulsory school for the years 2008–2013 according to the Swedish National School Register (N = 657,720). We linked the data to the Total Population Register, with information on death, birth country, identity of parents and migration^27^. We excluded children dying the month they graduated (n = 10), not born in Sweden (n = 55,794), missing identity of both parents (n = 13), and living abroad part of the nine year period prior to graduation (n = 9,361). We also excluded children with diagnoses of developmental delay or chromosomal deviations (n = 14,947) registered in the National Patient Register (NPR) according to the International Classification of Diseases, version 10 (ICD-10, codes: F70–F89, D82.1, Q87.1, Q87.8, Q90–Q93, Q98–99). The final cohort consisted of N = 570,595 children (Fig. 1).

The Regional Ethical Review Board in Stockholm, Sweden, approved the study (No. 2013/862–31/5) and allowed the researchers to waive the requirement for obtaining informed consent or parental permission. All data were pseudonymised and all methods were performed in accordance with the relevant guidelines and regulations (Good Clinical Practise and STROBE).

### Measures of asthma exposure

Asthma exposure was based on data from the Swedish Prescribed Drug Register (SPDR) and NPR. The SPDR started on July 1st 2005. It contains data on all prescribed drugs dispensed at Swedish pharmacies, coded according to the Anatomical Therapeutic Chemical classification system (ATC) with information on strength, package size and dates of prescription and dispense^28^. The NPR has full coverage of all inpatient hospital visits since 1987 and ~80% of all outpatient visits since 2001, with information on visit dates, diagnoses coded by the ICD system and whether the visit was planned or not^29^.

All asthma measures were defined for two time intervals based on their age at certain school Grades, namely Grades 7–8 and Grade 9. Each Grade was counted from July 1st the year the child started that grade until June 30th the following year. We created the cohort such that all children should have full coverage in the SPDR during Grades 7–8 and 9.

We defined asthma in accordance with a validation study^30^, as having, in the time interval, either of:

1. At least two dispenses of asthma control medication, i.e. inhaled corticosteroids, ICS (ATC: R03BA), leukotriene receptor antagonists (ATC: R03DC03) or combinations of ICS and β2 adrenergic receptor agonists (ATC: R03AK) or
2. Three dispenses of control medication and/or inhaled β2 adrenergic receptor agonists (ATC: R03AC02–03, R03AC12–13) in the SPDR or
3. A hospital visit with an asthma diagnosis recorded in the NPR.

We defined severe asthma to align with criteria in the Global Initiative for Asthma (GINA)^1^ as much as possible. For severe asthma within a time interval, we required a daily average dose of ICS above the medium dose limits for 12 years old children according to GINA, in combination with at least one other type of asthma control medication. We calculated the daily average dose as the total amount dispensed within the time interval divided by the number of days in the interval. Mild/moderate asthma was defined as asthma not being classified as severe.

### Measures of school performance

In Sweden, most children start school the year they turn seven. The first nine years of school are compulsory. After Grade 9, the students can apply to upper secondary school (USS), based on their grade point sum in Grade 9. The *grade point sum* is the sum of 16 subject grades. The highest possible grade in a subject gives 20 points, passing 10 points and failing 0 points, resulting in a range from 0 to 320 points. Children are *non-eligible for USS* if not attaining a passing grade in each of the core subjects: Swedish, mathematics and English.

In Grade 9, the students take national tests in Swedish, mathematics and English. The results range from 0–20. A more thorough description of the Swedish school system has been provided by Jangmo *et al*.^20^.

### Covariates

We selected covariates based on prior knowledge and a directed acyclic graph. Thus, we included gender, birth month (January-December) and ADHD. We defined ADHD as having either an ADHD diagnosis in the NPR (ICD-10: F90) or at least one dispensed ADHD medication in the SPDR (ATC codes: N06BA01, N06BA04, N06BA09), as previously validated^31^.

We collected the parents’ birth countries (Sweden, other Nordic country, European Union outside the Nordic countries (EU), Europe outside EU, Africa, North America/Oceania, South America and Asia) from the Total Population Register. As measures of parental socio-economic status, we retrieved information on the parents’ education level (middle school <9 years, middle school 9 years, upper secondary school 1–2 years, upper secondary school 3 years, college/University <3 years, college/university ≥3 years and post-graduate education) at the time the child started school from the Longitudinal Integration Database for Health Insurance and Labour Market Studies (LISA by Swedish acronym). Further, we included the family’s disposable income the year the child started school from LISA, divided into quintiles. Families in the lowest income quintile had a disposable income 33% lower than median for the group and families in the highest income quintile at least 30% higher. Finally, parental asthma was defined as an asthma diagnosis in the NPR or at least two dispenses of asthma medication in the SPDR, any time before the child started Grade 9 of school.

### Statistical analysis

In the cohort, we estimated the associations between asthma exposures (asthma yes/no and severe/uncontrolled asthma) and *grade point sum* and *national test results* using linear regression models, while we used logistic regression for associations between asthma exposures and *non-eligibility for USS*. For linear regression models, we used robust standard errors since the residual distributions were slightly skewed. For each outcome-exposure combination, we ran both unadjusted models and models adjusted for all our covariates. We also stratified the analyses by ADHD and highest achieved parental education and tested for interaction using Wald test^32^.

For sibling comparison, we created all possible full sibling pairs within the family and estimated the associations between asthma and school performance using fixed effects linear regression for grade point sum and national test results and conditional logistic regression for non-eligibility for USS. The sibling comparisons control for all confounders the siblings share, including unmeasured confounders, such as genes and family environment. In addition, we adjusted those models for the covariates ADHD and family income the year the child started school. Only sibling pairs who are discordant for both asthma and school performance measure are informative for the estimates of interest. Therefore, those frequencies are given in the tables. We used cluster robust standard errors to account for the clustering within families.

For quantitative outcomes Cohen’s d = β/SD was estimated as a measure of effect size, where β = regression coefficient and SD = standard deviation in the study population. Cohen proposed a categorisation of effect size such that d ≥ 0.20 corresponds to a small effect, d ≥ 0.50 to a medium-sized effect, and d ≥ 0.80 to a large effect^33^. We used Stata IC version 15.1 for all analyses.

## Results

In the study population n = 31,173 (5.5%) children had asthma in Grades 7–8 (Tables 1) and 3.4% had asthma in Grade 9 (Table A2, Appendix). Of all children with asthma, 3.5% had severe asthma and 9.1% had uncontrolled asthma. Asthma overall was more common in boys, in children with ADHD and in families with higher education and income (Table 1). In contrast, uncontrolled asthma was more common in families with lower education and income. Distributions of child’s birth month and paternal factors by asthma in Grades 7–8 are found in Table A1 in Appendix. In the study population, 97.6% had full information on all covariates listed in Table 1 and Table A1 in Appendix.

**Table 1:** Descriptives of study population by asthma status in school Grades 7–8.

	No asthma		Mild/mod controlled		Severe controlled		Mild/mod uncontrolled		Severe uncontrolled	
	N	%	N	%	N	%	N	%	N	%
**Study population**	539,422	94.5	27,442	4.8	897	0.2	2,627	0.5	207	0.0
**Child’s gender**										
Boy	272,166	94.0	15,405	5.3	553	0.2	1,373	0.5	116	0.0
Girl	267,256	95.1	12,037	4.3	344	0.1	1,254	0.4	91	0.0
**Birth month**										
January	45,426	94.0	2,571	5.3	83	0.2	242	0.5	28	0.1
February	44,596	94.1	2,485	5.2	65	0.1	207	0.4	22	0.0
March	51,140	94.3	2,710	5.0	81	0.1	256	0.5	17	0.0
April	51,209	94.5	2,626	4.8	98	0.2	222	0.4	17	0.0
May	48,984	94.8	2,313	4.5	90	0.2	246	0.5	16	0.0
June	47,709	95.0	2,194	4.4	74	0.1	214	0.4	11	0.0
July	47,548	95.1	2,154	4.3	68	0.1	219	0.4	15	0.0
August	45,855	94.7	2,251	4.6	70	0.1	225	0.5	21	0.0
September	42,930	94.6	2,166	4.8	80	0.2	205	0.5	13	0.0
October	40,653	94.5	2,047	4.8	75	0.2	209	0.5	14	0.0
November	36,411	94.2	1,974	5.1	64	0.2	206	0.5	15	0.0
December	36,961	94.4	1,951	5.0	49	0.1	176	0.4	18	0.0
**ADHD**										
No	518,394	94.6	25,973	4.7	842	0.2	2,439	0.4	194	0.0
Yes	21,028	92.4	1,469	6.5	55	0.2	188	0.8	13	0.1
**Mother’s birth place**										
Sweden	460,671	94.3	24,418	5.0	826	0.2	2,256	0.5	183	0.0
Nordic country	15,336	94.6	781	4.8	12	0.1	70	0.4	8	0.0
EU	9,325	95.8	360	3.7	13	0.1	32	0.3	1	0.0
Europe, not EU	17,255	97.1	440	2.5	6	0.0	60	0.3	3	0.0
Africa	6,958	94.5	348	4.7	9	0.1	47	0.6	4	0.1
North America, Oceania	1,544	94.1	84	5.1	2	0.1	9	0.5	1	0.1
South America	4,165	94.2	207	4.7	4	0.1	43	1.0	1	0.0
Asia	24,148	96.2	804	3.2	25	0.1	110	0.4	6	0.0
Missing	20	100.0	0	0.0	0	0.0	0	0.0	0	0.0
**Father’s birth place**										
Sweden	454,320	94.4	24,016	5.0	812	0.2	2,193	0.5	185	0.0
Nordic country	14,130	94.6	701	4.7	21	0.1	77	0.5	8	0.1
EU	10,265	95.4	432	4.0	8	0.1	49	0.5	1	0.0
Europe, not EU	18,982	97.1	485	2.5	8	0.0	64	0.3	1	0.0
Africa	8,768	93.8	490	5.2	16	0.2	64	0.7	6	0.1
North America, Oceania	2,178	93.2	148	6.3	3	0.1	8	0.3	0	0.0
South America	4,358	94.1	224	4.8	4	0.1	43	0.9	0	0.0
Asia	23,727	96.2	805	3.3	21	0.1	108	0.4	5	0.0
Missing	2,694	94.2	141	4.9	4	0.1	21	0.7	1	0.0
**Mother’s education**										
Middle school <9 years	10,692	96.7	311	2.8	2	0.0	43	0.4	6	0.1
Middle school 9 years	58,228	95.2	2,542	4.2	69	0.1	336	0.5	14	0.0
Upper secondary school 1–2 years	196,062	94.4	10,251	4.9	317	0.2	1,077	0.5	90	0.0
Upper secondary school 3 years	97,016	94.6	4,877	4.8	178	0.2	449	0.4	28	0.0
College/University <3 years	96,392	94.1	5,349	5.2	192	0.2	432	0.4	35	0.0
College/University ≥ 3years	74,794	94.6	3,875	4.9	136	0.2	268	0.3	32	0.0
Post-graduate education	2,339	95.0	117	4.8	2	0.1	4	0.2	0	0.0
Missing	3,899	96.5	120	3.0	1	0.0	18	0.4	2	0.0
**Father’s education**										
Middle school <9 years	12,270	95.6	481	3.7	15	0.1	60	0.5	5	0.0
Middle school 9 years	74,559	94.8	3,570	4.5	88	0.1	426	0.5	25	0.0
Upper secondary school 1–2 years	219,417	94.4	11,472	4.9	381	0.2	1,136	0.5	86	0.0
Upper secondary school 3 years	69,312	94.6	3,473	4.7	115	0.2	303	0.4	29	0.0
College/University <3 years	77,328	94.3	4,138	5.0	172	0.2	359	0.4	33	0.0
College/University ≥ 3years	70,636	94.7	3537	4.7	108	0.1	265	0.4	24	0.0
Post-graduate education	6,379	95.4	284	4.2	7	0.1	19	0.3	1	0.0
Missing	9,521	94.4	487	4.8	11	0.1	59	0.6	4	0.0
**Disposable family income**										
1st Quintile	108,427	95.0	4,851	4.3	128	0.1	654	0.6	39	0.0
2nd Quintile	108,018	94.7	5,333	4.7	156	0.1	500	0.4	35	0.0
3rd Quintile	107,623	94.3	5,825	5.1	185	0.2	499	0.4	44	0.0
4th Quintile	107,603	94.2	5,776	5.1	205	0.2	541	0.5	50	0.0
5th Quintile	107,688	94.4	5,654	5.0	223	0.2	433	0.4	39	0.0
Missing	63	95.5	3	4.5	0	0.0	0	0.0	0	0.0
**Asthma in mother**										
No	448,547	95.3	19,730	4.2	605	0.1	1,860	0.4	134	0.0
Yes	90,875	91.1	7,712	7.7	292	0.3	767	0.8	73	0.1
**Asthma in father**										
No	473,073	95.0	22,047	4.4	686	0.1	2,105	0.4	160	0.0
Yes	66,349	91.5	5,395	7.4	211	0.3	522	0.7	47	0.1

The mean of the grade point sum was 213 points with SD = 62 points, and 8.9% of the children were non-eligible for USS. In the study population, 95%, 94% and 96% had national test results in English, mathematics and Swedish respectively. The mean national test results were 14.0 (SD = 4.2) in English, 11.0 (SD = 5.6) in mathematics and 12.9 (SD = 4.0) in Swedish.

### Grade point sum and non-eligibility for USS by asthma overall

Asthma overall was associated with somewhat better school performance (higher grade point sum, lower odds of non-eligibility for USS), irrespective of what time interval asthma was measured (Table 2). For example, asthma in Grades 7–8 was associated with β = 2.1 points higher mean grade point sum (95% CI: 1.4–2.8) in the unadjusted model and after adjustment for measured confounders β = 3.9 points higher grade point sum (95% CI: 3.3–4.5; Cohen’s d = 0.06). However, in the sibling comparisons, which additionally controlled for unmeasured confounding factors that the siblings shared, the differences were close to null, e.g. β = 0.4 (95% CI: −0.6 to 1.5) for asthma in Grades 7–8. The odds ratio (OR) for the association between asthma in Grades 7–8 and non-eligibility for USS was 0.84 (95% CI: 0.80–0.88) in the adjusted model. Again, the estimate was close to null when comparing siblings, OR = 0.99 (95% CI: 0.87–1.12).

**Table 2:** Associations of asthma (yes vs no) in different school years with grade point sum and non-eligibility to upper secondary school (USS).

Grade point sum	Unadjusted model			Adjusted model^a^				Sibling comparison^b^			
	n	β	[95% CI]	n	β	[95% CI]	Cohen’s d	n^c^	β	[95% CI]	Cohen’s d
Asthma in Grades 7–8	570,595	2.1	[1.4,2.8]	556,994	3.9	[3.3,4.5]	0.06	22,450	0.4	[−0.6,1.5]	0.01
Asthma in Grade 9	570,595	4.1	[3.3,5.0]	556,994	4.9	[4.2,5.7]	0.08	14,478	0.2	[−1.1,1.5]	0.00
Non-eligibility to USS	n	OR	[95% CI]	n	OR	[95% CI]		n^c^	OR	[95% CI]	
Asthma in Grades 7–8	570,595	0.86	[0.83,0.90]	556,994	0.84	[0.80,0.88]		2,354	0.99	[0.87,1.12]	
Asthma in Grade 9	570,595	0.85	[0.80,0.89]	556,994	0.84	[0.80,0.89]		1,352	1.07	[0.91,1.26]	

^a^Adjusted models were adjusted for gender, ADHD, gender x ADHD interaction, mother’s and father’s education and asthma, family income the year the child started school.

^b^Sibling comparisons were adjusted for all familial factors shared by the siblings + gender, ADHD, gender x ADHD interaction and family income the year the child started school.

^c^Number of children in pairs discordant for both asthma variable and school performance measure.

### Grade point sum and non-eligibility for USS by asthma control and severity

Children with mild/moderate uncontrolled asthma showed *lower* school performance, while children with severe controlled asthma performed slightly *better* compared to children without asthma (Table 3). For example, severe controlled asthma in Grades 7–8 was associated with β = 12.5 points *higher* grade point sum (95% CI: 9.3–15.7; Cohen’s d = 0.20), adjusted for measured confounders. In contrast, mild/moderate uncontrolled asthma in Grades 7–8 was associated with 6.7 points *lower* grade point sum in adjusted models (β = −6.7 95% CI: −8.9 to −4.5; Cohen’s d = 0.11) and 9.9 points lower if having mild/moderate uncontrolled asthma in Grade 9 (β = −9.9 95% CI: −12.8 to −7.0; Cohen’s d = 0.16). In the sibling comparisons, most estimates were attenuated and close to the null. However, the association between severe controlled asthma in Grades 7–8 and grade point sum remained at β = 7.3 points *higher* grade point sum (95% CI: 2.3–15.7; Cohen’s d = 0.12) and mild/moderate uncontrolled asthma in Grade 9 was associated with 7.6 points *lower* grade point sum with (β = −7.6 points, 95% CI: −12.6 to −2.6; Cohen’s d = 0.12).

**Table 3:** Associations of asthma status in different Grades in school with grade point sum and non-eligibility to upper secondary school (USS).

Grade point sum	Unadjusted model			Adjusted model^**a**^				Sibling comparison^**b**^			
	n	β	[95% CI]	n	β	[95% CI]	Cohen’s d	n^**c**^	β	[95% CI]	Cohen’s d
**Asthma in Grades 7–8**											
No	539,422	0.0		526,503	0.0			11,225	0.0		
Mild/moderate controlled	27,442	3.4	[2.6, 4.1]	26,852	4.6	[4.0, 5.3]	0.07	10,120	0.3	[−0.8, 1.4]	0.00
Severe controlled	897	13.0	[9.2, 16.7]	885	12.5	[9.3, 15.7]	0.20	403	7.3	[2.3, 12.3]	0.12
Mild/moderate uncontrolled	2,627	−14.2	[−16.7, −11.7]	2,553	−6.7	[−8.9, −4.5]	0.11	1,086	−0.5	[−3.9, 2.9]	0.01
Severe uncontrolled	207	0.6	[−8.6, 9.7]	201	4.5	[−3.1, 12.2]	0.07	84	2.9	[−10.2, 15.9]	0.05
**Asthma in Grade 9**											
No	551,128	0.0		537,931	0.0			7,239	0.0		
Mild/moderate controlled	16,281	5.8	[4.8, 6.7]	15,957	6.1	[5.3, 6.9]	0.10	6,210	1.0	[−0.4, 2.3]	0.02
Severe controlled	1,294	12.1	[8.9, 15.4]	1,270	10.3	[7.6, 13.0]	0.17	553	1.0	[−3.9, 5.9]	0.02
Mild/moderate uncontrolled	1,657	−17.2	[−20.6, −13.8]	1,605	−9.9	[−12.8, −7.0]	0.16	638	−7.6	[−12.6, −2.6]	0.12
Severe uncontrolled	235	−3.7	[−11.7, 4.3]	231	−2.9	[−9.8, 3.9]	0.05	110	−2.7	[−13.8, 8.4]	0.04
**Non-eligibility to USS**	**n**	**OR**	**[95% CI]**	**n**	**OR**	**[95% CI]**		**n**^**c**^	**OR**	**[95% CI]**	
**Asthma in Grades 7–8**											
No	539,422	1.00		526,503	1.00			1,177	1.00		
Mild/moderate controlled	27,442	0.82	[0.78, 0.86]	26,852	0.81	[0.77, 0.85]		1,031	1.03	[0.90, 1.18]	
Severe controlled	897	0.62	[0.47, 0.83]	885	0.68	[0.51, 0.91]		26	0.71	[0.28, 1.77]	
Mild/moderate uncontrolled	2,627	1.40	[1.25, 1.58]	2,553	1.15	[1.01, 1.30]		155	0.73	[0.51, 1.05]	
Severe uncontrolled	207	1.03	[0.64, 1.64]	201	0.87	[0.51, 1.48]		9	1.75	[0.56, 5.43]	
**Asthma in Grade 9**											
No	551,128	1.00		537,931	1.00			676	1.00		
Mild/moderate controlled	16,281	0.77	[0.72, 0.82]	15,957	0.78	[0.73, 0.83]		540	0.95	[0.79, 1.14]	
Severe controlled	1,294	0.73	[0.59, 0.91]	1,270	0.81	[0.64, 1.02]		52	1.05	[0.58, 1.91]	
Mild/moderate uncontrolled	1,657	1.75	[1.53, 2.00]	1,605	1.44	[1.23, 1.67]		96	1.96	[1.22, 3.14]	
Severe uncontrolled	235	1.00	[0.64, 1.56]	231	0.93	[0.57, 1.51]		10	2.67	[0.59, 12.05]	

^a^Adjusted models were adjusted for gender, ADHD, gender x ADHD interaction, mother’s and father’s education and asthma, family income the year the child started school.

^b^Sibling comparisons were adjusted for all familial factors shared by the siblings + gender, ADHD, gender x ADHD interaction and family income the year the child started school.

^c^Number of children in pairs discordant for both asthma variable and school performance measure.

We observed a similar pattern between controlled and uncontrolled asthma for non-eligibility for USS (Table 3). Controlled asthma was associated with a smaller risk of being non-eligible, while uncontrolled asthma was associated with a higher risk of being non-eligible. Again, in the sibling comparisons, a higher risk remained mainly in association with mild/moderate uncontrolled asthma in Grade 9 with OR = 1.96 (95% CI: 1.22–3.14).

### National test results by asthma control and severity

Results for the association between asthma status in Grade 9 and national test results in the same year (Table 4) showed slightly better school performance among children with severe controlled asthma compared to children without asthma, with differences ranging from null for English to 0.7 points (95% CI: 0.4–1.0; Cohen’s d = 0.17) for mathematics. In contrast, children with mild/moderate uncontrolled asthma had slightly lower test results than children without asthma, with 0.2–0.5 points lower means (Cohen’s d: 0.06–0.10). In the sibling comparisons, most differences decreased for children with controlled asthma, but remained for those with uncontrolled asthma.

**Table 4:** Association between asthma status in Grade 9 and results from national tests (range: 0–20 points) the same year.

	Unadjusted model			Adjusted model^a^				Sibling comparison^b^			
	n	β	[95% CI]	n	β	[95% CI]	Cohen’s d	n^c^	β	[95% CI]	Cohen’s d
**English**											
No asthma	525,295	0.0		513,306	0.0			3,969	0.0		
Mild/moderate controlled	15,587	0.1	[0.0, 0.2]	15,286	0.1	[0.0, 0.1]	0.02	3,423	−0.1	[−0.2, 0.0]	0.02
Severe controlled	1,231	0.2	[0.0, 0.5]	1,208	0.0	[−0.2, 0.3]	0.01	284	−0.2	[−0.6, 0.2]	0.05
Mild/moderate uncontrolled	1,494	−0.7	[−0.9,−0.5]	1,448	−0.4	[−0.6, −0.2]	0.10	342	−0.5	[−0.9, −0.1]	0.12
Severe uncontrolled	212	−0.5	[−1.0, −0.0]	209	−0.5	[−1.0, −0.1]	0.12	58	−0.3	[−1.2, 0.6]	0.07
**Mathematics**											
No asthma	517,470	0.0		505,846	0.0			4,161	0.0		
Mild/moderate controlled	15,397	0.4	[0.3, 0.5]	15,101	0.3	[0.2, 0.4]	0.06	3,608	0.0	[−0.1, 0.2]	0.01
Severe controlled	1,215	1.0	[0.7, 1.3]	1,193	0.7	[0.4, 1.0]	0.12	330	0.2	[−0.3, 0.7]	0.04
Mild/moderate uncontrolled	1,486	−0.9	[−1.2, −0.6]	1,444	−0.5	[−0.8, −0.2]	0.09	316	−0.3	[−0.9, 0.2]	0.06
Severe uncontrolled	211	−0.8	[−1.5, 0.0]	208	−0.6	[−1.3, 0.1]	0.11	59	−0.3	[−1.6, 1.1]	0.05
**Swedish**											
No asthma	529,382	0.0		517,277	0.0			4,077	0.0		
Mild/moderate controlled	15,691	0.2	[0.1, 0.3]	15,388	0.2	[0.2, 0.3]	0.06	3,533	−0.0	[−0.1, 0.1]	0.00
Severe controlled	1,237	0.5	[0.3, 0.7]	1,214	0.4	[0.2, 0.6]	0.10	305	0.0	[−0.3, 0.4]	0.01
Mild/moderate uncontrolled	1,546	−0.4	[−0.6, −0.2]	1,501	−0.2	[−0.4, −0.1]	0.06	340	−0.3	[−0.7, 0.0]	0.08
Severe uncontrolled	220	−0.1	[−0.6, 0.4]	217	−0.2	[−0.6, 0.3]	0.05	55	−0.4	[−1.2, 0.4]	0.11

^a^ Adjusted models were adjusted for gender, ADHD, gender x ADHD interaction, mother’s and father’s education and asthma, family income the year the child started school.

^b^ Sibling comparisons were adjusted for all familial factors shared by the siblings + gender, ADHD, gender x ADHD interaction and family income the year the child started school.

^c^ Number of children in pairs discordant for both asthma variable and school performance measure.

### Stratification by ADHD and parental education

Analyses stratified by ADHD showed no major differences (Table A3, Appendix), while stratifying by parental education showed stronger associations between uncontrolled asthma and poorer school performance with lower parental education (Table A4, Appendix).

## Discussion

In analyses that were unadjusted or adjusted for measured confounders, children with asthma and severe controlled asthma in Grades 7–8 and 9 appeared to have slightly better grade point sums in Grade 9, a slightly lower risk of not being eligible for upper secondary school and slightly better results on national tests than children without asthma. In contrast, children with uncontrolled asthma appeared to have poorer results for all measures of school performance. However, most estimates were attenuated in the sibling comparisons, which controls for all genetic and environmental confounders that the siblings share, such as parent characteristics, home environment and family life style, but not characteristics that are unique for each sibling. The only associations that consistently remained in the sibling comparisons were those between uncontrolled asthma in Grade 9 and poorer school performance (grade point sum, non-eligibility for upper secondary school and national test in English). Our results are novel and further our understanding of the association between asthma and school performance.

Results from previous studies are mixed, some showing lower performance in children with asthma compared to other children^6,8,9,13,34–37^ and others showing no difference or better performance^14,25,38–41^. If the association between asthma and school performance depends on the level of asthma severity and control, as suggested by our results, differences in asthma definitions could explain variances in previous studies. Most have included children in rather wide age ranges, often from primary up to upper secondary school^6,8,9,34,35,38–40^. It is possible that a detrimental effect of asthma on school performance could vary by age. There is some evidence of lower school readiness among children with asthma when beginning kindergarten^42^. Thus, differences in the age distribution of the study populations could also explain variation in results between previous studies. Tsakiri *et al*.^8^ distinguished between grade point average from elementary school and middle school. In elementary school, the children with asthma had lower grades than other children, but not in middle school. Two other studies, both from Sweden, on the association between asthma and grades point sum from Grade 9, found lower grades among children with asthma^13^ and no differences^14^, respectively. It should be noted that both studies were much smaller than the present study and the one finding lower grades among children with asthma had only 42% response rate, with non-response associated with parental socio-economic status, gender and asthma. There is one previous small study with sibling comparisons, which in line with our results, saw no differences in school performance between the children treated for asthma and their non-asthmatic siblings^25^.

The attenuation of the estimates, when controlling for all confounders that siblings share, indicates that the reason why children with severe controlled asthma performed better in school while the children with uncontrolled asthma performed worse could be confounding by familial factors, genetic or environmental, that affect both the child’s asthma control and school performance, e.g. aspects of parental education not captured by formal education. To speculate, the fact that it remained for uncontrolled asthma in Grade 9, when the individual is 15–16 years old, but not at lower ages, could be explained by higher influence of the adolescent’s own characteristics on his/her medication compared to younger children^43^. If that is true, we cannot rule out the possibility that also the estimates from the sibling comparisons between asthma control at that age and school performance were confounded, e.g. by personal traits, such as compliance to asthma treatment regime and school requirements or other factors that affects both the adolescent’s tendency to medicate properly and his/her school performance.

Considering that ADHD comorbidity and parental socio-economic status could play an important role in the associations, we also performed analyses stratified by those factors. When stratifying on parental education, children with uncontrolled asthma were more disadvantaged if their parents had no more than middle school compared to those with more educated parents. No differences were seen with regard to ADHD.

Most of the associations we found seemed to be due to confounding from familial factors, rather than being a causal effect of asthma on school performance. However, if there is a causal effect due to uncontrolled asthma it should be noted that it is small. The difference in grade point sum between children with uncontrolled asthma and those without asthma in Grade 9 was 7.6 points, corresponding to a Cohen’s d of 0.12, as compared to differences found between children with and without ADHD, which in another study has been estimated to 56 points^20^.

The strengths of this study include the use of register data with very low proportion of missing values, which makes it generalizable to settings with similar health care and school systems. The data was recorded prospectively, precluding recall bias. We had both information on grade point sum, which can be seen as a measure of overall school performance over a longer time period and results from national tests which mirrors the student’s performance at a single occasion. The large study population with information on family relations enabled us to conduct sibling comparisons, which are informative with regard to confounding from shared familial factors such as genes and life style that are difficult to measure.

Our study also has some limitations. We lacked information from general practitioners and thereby the mildest cases of asthma, not requiring treatment by an asthma specialist and with minimal usage of asthma medications, were misclassified as non-asthma cases. However, such mild asthma is unlikely to affect school performance. We also lacked information on worsening of asthma such as exacerbations with coughing, increased symptoms and other clinical features of the asthma as well as school absenteeism. Sibling comparisons also has some limitations^24^. One important limitation is an increased sensitivity to measurement error, in particular in the exposure variables, which may result in attenuated association estimates^44^. However, although the measures of asthma severity and control lacks validation, the algorithm for identifying children with asthma has shown high validity^30^. Despite the large study population, we had very few siblings who were discordant for severe uncontrolled asthma, giving very wide confidence intervals for some of the estimates in the sibling comparisons. Finally, there is no available information on school performance before Grade 9 so we could not study changes over time. To understand if there is a causal effect of uncontrolled asthma on school performance, school performance would need to be added as an outcome in a randomised controlled trials on measures to improve asthma control in teenagers.

In conclusion, we found a weak association between asthma in general and better school performance, whereas children with uncontrolled asthma showed slightly poorer school performance, which was possibly worse in children of parents with the lowest level of education. Most associations were clearly attenuated when controlling for familial factors using sibling comparisons, suggesting that the differences may be due to familial factors that affect both the child’s asthma control and his/her school performance. However, there remained an association between uncontrolled asthma in Grade 9 and several of the measures of school performance. This is consistent with a causal interpretation, but we cannot preclude confounding by factors not shared by siblings, e.g. personal traits that affects both the asthma control and the school performance. These findings are of great importance for understanding the associations seen between asthma and school performance.

## Supplementary information

Supplementary Information.
