# Supplementary material for: Asthma and subsequent school performance at age 15–16 years: A Swedish population-based sibling control study
Source: Sci Rep. 2020 May 6;10:7661. doi: 10.1038/s41598-020-64633-w (PMC7203156; doi:10.1038/s41598-020-64633-w)
Supplement: Supplementary file 1 — Supplementary Information. [file 41598_2020_64633_MOESM1_ESM.docx]

**Appendix**

Asthma and subsequent school performance at age 15-16 years: A Swedish population-based sibling control study

Cecilia Lundholm, Bronwyn Brew, Brian M D’Onofrio, Emma Caffrey Osvald, Henrik Larsson, Catarina Almqvist

**Table of Contents**

[**Table A1.** Descriptives of study population by asthma status in school Grades 7-8 2](#_Toc30770658)

[**Table A1.** Descriptives of study population by asthma status in school Grade 9 4](#_Toc30770659)

[**Table A2.** Association between asthma and grade point sum/non-eligibility to upper secondary school by ADHD status 6](#_Toc30770660)

[**Table A3.** Association between asthma and grade point sum/non-eligibility to upper secondary school by highest parental education 7](#_Toc30770661)

## **Table A1.** Descriptives of study population by asthma status in school Grades 7-8

|  | **No asthma** | | **Mild/mod controlled** | | **Severe controlled** | | **Mild/mod uncontrolled** | | **Severe uncontrolled** | |
| --- | --- | --- | --- | --- | --- | --- | --- | --- | --- | --- |
|  | N | % | N | % | N | % | N | % | N | % |
| **Study population** | 539,422 | 94.5 | 27,442 | 4.8 | 897 | 0.2 | 2,627 | 0.5 | 207 | 0.0 |
|  |  |  |  |  |  |  |  |  |  |  |
| **Child’s birth month** |  |  |  |  |  |  |  |  |  |  |
| January | 45,426 | 94.0 | 2,571 | 5.3 | 83 | 0.2 | 242 | 0.5 | 28 | 0.1 |
| February | 44,596 | 94.1 | 2,485 | 5.2 | 65 | 0.1 | 207 | 0.4 | 22 | 0.0 |
| March | 51,140 | 94.3 | 2,710 | 5.0 | 81 | 0.1 | 256 | 0.5 | 17 | 0.0 |
| April | 51,209 | 94.5 | 2,626 | 4.8 | 98 | 0.2 | 222 | 0.4 | 17 | 0.0 |
| May | 48,984 | 94.8 | 2,313 | 4.5 | 90 | 0.2 | 246 | 0.5 | 16 | 0.0 |
| June | 47,709 | 95.0 | 2,194 | 4.4 | 74 | 0.1 | 214 | 0.4 | 11 | 0.0 |
| July | 47,548 | 95.1 | 2,154 | 4.3 | 68 | 0.1 | 219 | 0.4 | 15 | 0.0 |
| August | 45,855 | 94.7 | 2,251 | 4.6 | 70 | 0.1 | 225 | 0.5 | 21 | 0.0 |
| September | 42,930 | 94.6 | 2,166 | 4.8 | 80 | 0.2 | 205 | 0.5 | 13 | 0.0 |
| October | 40,653 | 94.5 | 2,047 | 4.8 | 75 | 0.2 | 209 | 0.5 | 14 | 0.0 |
| November | 36,411 | 94.2 | 1,974 | 5.1 | 64 | 0.2 | 206 | 0.5 | 15 | 0.0 |
| December | 36,961 | 94.4 | 1,951 | 5.0 | 49 | 0.1 | 176 | 0.4 | 18 | 0.0 |
|  |  |  |  |  |  |  |  |  |  |  |
| **Father's birth place** |  |  |  |  |  |  |  |  |  |  |
| Sweden | 454,320 | 94.4 | 24,016 | 5.0 | 812 | 0.2 | 2,193 | 0.5 | 185 | 0.0 |
| Nordic country | 14,130 | 94.6 | 701 | 4.7 | 21 | 0.1 | 77 | 0.5 | 8 | 0.1 |
| EU | 10,265 | 95.4 | 432 | 4.0 | 8 | 0.1 | 49 | 0.5 | 1 | 0.0 |
| Europe, not EU | 18,982 | 97.1 | 485 | 2.5 | 8 | 0.0 | 64 | 0.3 | 1 | 0.0 |
| Africa | 8,768 | 93.8 | 490 | 5.2 | 16 | 0.2 | 64 | 0.7 | 6 | 0.1 |
| North America, Oceania | 2,178 | 93.2 | 148 | 6.3 | 3 | 0.1 | 8 | 0.3 | 0 | 0.0 |
| South America | 4,358 | 94.1 | 224 | 4.8 | 4 | 0.1 | 43 | 0.9 | 0 | 0.0 |
| Asia | 23,727 | 96.2 | 805 | 3.3 | 21 | 0.1 | 108 | 0.4 | 5 | 0.0 |
| Missing | 2,694 | 94.2 | 141 | 4.9 | 4 | 0.1 | 21 | 0.7 | 1 | 0.0 |
|  |  |  |  |  |  |  |  |  |  |  |
| **Father's education** |  |  |  |  |  |  |  |  |  |  |
| Middle school <9 years | 12,270 | 95.6 | 481 | 3.7 | 15 | 0.1 | 60 | 0.5 | 5 | 0.0 |
| Middle school 9 years | 74,559 | 94.8 | 3,570 | 4.5 | 88 | 0.1 | 426 | 0.5 | 25 | 0.0 |
| Upper secondary school 1-2 years | 219,417 | 94.4 | 11,472 | 4.9 | 381 | 0.2 | 1,136 | 0.5 | 86 | 0.0 |
| Upper secondary school 3 years | 69,312 | 94.6 | 3,473 | 4.7 | 115 | 0.2 | 303 | 0.4 | 29 | 0.0 |
| College/University < 3 years | 77,328 | 94.3 | 4,138 | 5.0 | 172 | 0.2 | 359 | 0.4 | 33 | 0.0 |
| College/University ≥ 3years | 70,636 | 94.7 | 3537 | 4.7 | 108 | 0.1 | 265 | 0.4 | 24 | 0.0 |
| Post-graduate education | 6,379 | 95.4 | 284 | 4.2 | 7 | 0.1 | 19 | 0.3 | 1 | 0.0 |
| Missing | 9,521 | 94.4 | 487 | 4.8 | 11 | 0.1 | 59 | 0.6 | 4 | 0.0 |
|  |  |  |  |  |  |  |  |  |  |  |
| **Asthma in father** |  |  |  |  |  |  |  |  |  |  |
| No | 473,073 | 95.0 | 22,047 | 4.4 | 686 | 0.1 | 2,105 | 0.4 | 160 | 0.0 |
| Yes | 66,349 | 91.5 | 5,395 | 7.4 | 211 | 0.3 | 522 | 0.7 | 47 | 0.1 |

## **Table A2.** Descriptives of study population by asthma status in school Grade 9

|  | **No asthma** | | **Mild/mod controlled** | | **Severe controlled** | | **Mild/mod uncontrolled** | | **Severe un-controlled** | |
| --- | --- | --- | --- | --- | --- | --- | --- | --- | --- | --- |
|  | N | % | N | % | N | % | N | % | N | % |
| **Study population** | 551,128 | 96.6 | 16,281 | 2.9 | 1,294 | 0.2 | 1,657 | 0.3 | 235 | 0.0 |
|  |  |  |  |  |  |  |  |  |  |  |
| **Child's gender** |  |  |  |  |  |  |  |  |  |  |
| Boy | 279,244 | 96.4 | 8,781 | 3.0 | 733 | 0.3 | 750 | 0.3 | 105 | 0.0 |
| Girl | 271,884 | 96.8 | 7l500 | 2.7 | 561 | 0.2 | 907 | 0.3 | 130 | 0.0 |
|  |  |  |  |  |  |  |  |  |  |  |
| **Birth month** |  |  |  |  |  |  |  |  |  |  |
| January | 46,487 | 96.1 | 1,534 | 3.2 | 127 | 0.3 | 180 | 0.4 | 22 | 0.0 |
| February | 45,618 | 96.3 | 1,478 | 3.1 | 133 | 0.3 | 130 | 0.3 | 16 | 0.0 |
| March | 52,238 | 96.4 | 1,623 | 3.0 | 131 | 0.2 | 176 | 0.3 | 36 | 0.1 |
| April | 52,300 | 96.5 | 1,582 | 2.9 | 123 | 0.2 | 147 | 0.3 | 20 | 0.0 |
| May | 49,993 | 96.8 | 1,401 | 2.7 | 96 | 0.2 | 133 | 0.3 | 26 | 0.1 |
| June | 48,726 | 97.1 | 1,248 | 2.5 | 97 | 0.2 | 115 | 0.2 | 16 | 0.0 |
| July | 48,451 | 96.9 | 1,306 | 2.6 | 111 | 0.2 | 126 | 0.3 | 10 | 0.0 |
| August | 46,810 | 96.7 | 1,335 | 2.8 | 116 | 0.2 | 136 | 0.3 | 25 | 0.1 |
| September | 43,869 | 96.6 | 1,285 | 2.8 | 89 | 0.2 | 134 | 0.3 | 17 | 0.0 |
| October | 41,503 | 96.5 | 1,258 | 2.9 | 96 | 0.2 | 127 | 0.3 | 14 | 0.0 |
| November | 37,317 | 96.5 | 1,105 | 2.9 | 107 | 0.3 | 121 | 0.3 | 20 | 0.1 |
| December | 37,816 | 96.6 | 1,126 | 2.9 | 68 | 0.2 | 132 | 0.3 | 13 | 0.0 |
|  |  |  |  |  |  |  |  |  |  |  |
| **ADHD** |  |  |  |  |  |  |  |  |  |  |
| No | 529,431 | 96.6 | 15,462 | 2.8 | 1,216 | 0.2 | 1,508 | 0.3 | 225 | 0.0 |
| Yes | 21,697 | 95.4 | 819 | 3.6 | 78 | 0.3 | 149 | 0.7 | 10 | 0.0 |
|  |  |  |  |  |  |  |  |  |  |  |
| **Mother's birth place** |  |  |  |  |  |  |  |  |  |  |
| Sweden | 470,870 | 96.4 | 14,638 | 3.0 | 1,181 | 0.2 | 1,454 | 0.3 | 211 | 0.0 |
| Nordic country | 15,693 | 96.8 | 439 | 2.7 | 27 | 0.2 | 46 | 0.3 | 2 | 0.0 |
| EU | 9,478 | 97.4 | 200 | 2.1 | 19 | 0.2 | 29 | 0.3 | 5 | 0.1 |
| Europe, not EU | 17,506 | 98.5 | 217 | 1.2 | 9 | 0.1 | 30 | 0.2 | 2 | 0.0 |
| Africa | 7,135 | 96.9 | 183 | 2.5 | 21 | 0.3 | 22 | 0.3 | 5 | 0.1 |
| North America, Oceania | 1,583 | 96.5 | 42 | 2.6 | 5 | 0.3 | 9 | 0.5 | 1 | 0.1 |
| South America | 4,278 | 96.8 | 116 | 2.6 | 7 | 0.2 | 17 | 0.4 | 2 | 0.0 |
| Asia | 24,566 | 97.9 | 445 | 1.8 | 25 | 0.1 | 50 | 0.2 | 7 | 0.0 |
| Missing | 19 | 95.0 | 1 | 5.0 | 0 | 0.0 | 0 | 0.0 | 0 | 0.0 |
|  |  |  |  |  |  |  |  |  |  |  |
| **Father's birth place** |  |  |  |  |  |  |  |  |  |  |
| Sweden | 464,388 | 96.4 | 14,328 | 3.0 | 1,172 | 0.2 | 1,432 | 0.3 | 206 | 0.0 |
| Nordic country | 14,392 | 96.4 | 455 | 3.0 | 28 | 0.2 | 54 | 0.4 | 8 | 0.1 |
| EU | 10,462 | 97.3 | 251 | 2.3 | 11 | 0.1 | 30 | 0.3 | 1 | 0.0 |
| Europe, not EU | 19,242 | 98.5 | 254 | 1.3 | 11 | 0.1 | 32 | 0.2 | 1 | 0.0 |
| Africa | 9,014 | 96.5 | 263 | 2.8 | 27 | 0.3 | 30 | 0.3 | 10 | 0.1 |
| North America, Oceania | 2,259 | 96.7 | 69 | 3.0 | 5 | 0.2 | 3 | 0.1 | 1 | 0.0 |
| South America | 4,479 | 96.8 | 127 | 2.7 | 8 | 0.2 | 13 | 0.3 | 2 | 0.0 |
| Asia | 24,139 | 97.9 | 448 | 1.8 | 25 | 0.1 | 49 | 0.2 | 5 | 0.0 |
| Missing | 2,753 | 96.2 | 86 | 3.0 | 7 | 0.2 | 14 | 0.5 | 1 | 0.0 |
|  |  |  |  |  |  |  |  |  |  |  |
| **Mother's education** |  |  |  |  |  |  |  |  |  |  |
| Middle school <9 years | 10,862 | 98.3 | 161 | 1.5 | 6 | 0.1 | 21 | 0.2 | 4 | 0.0 |
| Middle school 9 years | 59,416 | 97.1 | 1,455 | 2.4 | 83 | 0.1 | 210 | 0.3 | 25 | 0.0 |
| Upper secondary school 1-2 years | 200,429 | 96.5 | 6,150 | 3.0 | 458 | 0.2 | 661 | 0.3 | 99 | 0.0 |
| Upper secondary school 3 years | 99,092 | 96.6 | 2,890 | 2.8 | 231 | 0.2 | 295 | 0.3 | 40 | 0.0 |
| College/University < 3 years | 98,569 | 96.3 | 3,233 | 3.2 | 285 | 0.3 | 273 | 0.3 | 40 | 0.0 |
| College/University ≥ 3years | 76,397 | 96.6 | 2,285 | 2.9 | 222 | 0.3 | 176 | 0.2 | 25 | 0.0 |
| Post-graduate education | 2,404 | 97.6 | 46 | 1.9 | 4 | 0.2 | 7 | 0.3 | 1 | 0.0 |
| Missing | 3,959 | 98.0 | 61 | 1.5 | 5 | 0.1 | 14 | 0.3 | 1 | 0.0 |
|  |  |  |  |  |  |  |  |  |  |  |
| **Father's education** |  |  |  |  |  |  |  |  |  |  |
| Middle school <9 years | 12,501 | 97.4 | 276 | 2.2 | 18 | 0.1 | 33 | 0.3 | 3 | 0.0 |
| Middle school 9 years | 76,142 | 96.8 | 2,059 | 2.6 | 146 | 0.2 | 292 | 0.4 | 29 | 0.0 |
| Upper secondary school 1-2 years | 224,249 | 96.5 | 6,886 | 3.0 | 524 | 0.2 | 727 | 0.3 | 106 | 0.0 |
| Upper secondary school 3 years | 70,784 | 96.7 | 2,058 | 2.8 | 165 | 0.2 | 193 | 0.3 | 32 | 0.0 |
| College/University < 3 years | 79,097 | 96.4 | 2,465 | 3.0 | 219 | 0.3 | 213 | 0.3 | 36 | 0.0 |
| College/University ≥ 3years | 72,095 | 96.7 | 2,115 | 2.8 | 192 | 0.3 | 145 | 0.2 | 23 | 0.0 |
| Post-graduate education | 6,512 | 97.3 | 151 | 2.3 | 10 | 0.1 | 14 | 0.2 | 3 | 0.0 |
| Missing | 9,748 | 96.7 | 271 | 2.7 | 20 | 0.2 | 40 | 0.4 | 3 | 0.0 |
|  |  |  |  |  |  |  |  |  |  |  |
| **Disposable family income** |  |  |  |  |  |  |  |  |  |  |
| 1st Quintile | 110,740 | 97.1 | 2,750 | 2.4 | 189 | 0.2 | 376 | 0.3 | 44 | 0.0 |
| 2nd Quintile | 110,273 | 96.7 | 3,152 | 2.8 | 240 | 0.2 | 342 | 0.3 | 35 | 0.0 |
| 3rd Quintile | 110,022 | 96.4 | 3,482 | 3.0 | 270 | 0.2 | 347 | 0.3 | 55 | 0.0 |
| 4th Quintile | 109,964 | 96.3 | 3,524 | 3.1 | 307 | 0.3 | 324 | 0.3 | 56 | 0.0 |
| 5th Quintile | 110,064 | 96.5 | 3,372 | 3.0 | 288 | 0.3 | 268 | 0.2 | 45 | 0.0 |
| Missing | 65 | 98.5 | 1 | 1.5 | 0 | 0.0 | 0 | 0.0 | 0 | 0.0 |
|  |  |  |  |  |  |  |  |  |  |  |
| **Asthma in mother** |  |  |  |  |  |  |  |  |  |  |
| No | 457,002 | 97.1 | 11,703 | 2.5 | 885 | 0.2 | 1,137 | 0.2 | 149 | 0.0 |
| Yes | 94,126 | 94.4 | 4,578 | 4.6 | 409 | 0.4 | 520 | 0.5 | 86 | 0.1 |
|  |  |  |  |  |  |  |  |  |  |  |
| **Asthma in father** |  |  |  |  |  |  |  |  |  |  |
| No | 482,601 | 96.9 | 12,984 | 2.6 | 992 | 0.2 | 1,315 | 0.3 | 179 | 0.0 |
| Yes | 68,527 | 94.5 | 3,297 | 4.5 | 302 | 0.4 | 342 | 0.5 | 56 | 0.1 |

## **Table A3.** Association between asthma and grade point sum/non-eligibility to upper secondary school by ADHD status

|  | **No ADHD** | | | **ADHD** | | |
| --- | --- | --- | --- | --- | --- | --- |
|  | **Adjusted^a^** | | | **Adjusted^a^** | | |
| **Grade point sum** | **n** | **β** | **[95% CI]** | **n** | **β** | **[95% CI]** |
| Asthma in Grades 7-8 | 535,049 | 3.9 | [3.3,4.5] | 21,945 | 4.8 | [1.4,8.1] |
| Asthma in Grade 9 | 535,049 | 4.9 | [4.1,5.6] | 21,945 | 5.6 | [1.3,9.9] |
|  |  |  |  |  |  |  |
| **Non-eligibility to upper secondary school** | **n** | **OR** | **[95% CI]** | **n** | **OR** | **[95% CI]** |
| Asthma in Grades 7-8 | 535,049 | 0.84 | [0.80,0.88] | 21,945 | 0.82 | [0.73,0.92] |
| Asthma in Grade 9 | 535,049 | 0.84 | [0.79,0.90] | 21,945 | 0.87 | [0.75,1.00] |
|  |  |  |  |  |  |  |
| **Grade point sum** | **n** | **β** | **[95% CI]** | **n** | **β** | **[95% CI]** |
| **Asthma in Grades 7-8** |  |  |  |  |  |  |
| No | 506,210 | 0.0 |  | 20,293 | 0.0 |  |
| Mild/moderate controlled | 25,442 | 4.5 | [3.9,5.2] | 1,410 | 6.3 | [2.8,9.9] |
| Severe controlled | 832 | 12.3 | [9.2,15.5] | 53 | 15.7 | [-5.3,36.6] |
| Mild/moderate uncontrolled | 2,377 | -6.5 | [-8.7,-4.3] | 176 | -9.3 | [-20.2,1.6] |
| Severe uncontrolled | 188 | 6.1 | [-1.4,13.5] | 13 | -19.1 | [-66.0,27.8] |
|  |  |  |  |  |  |  |
| **Asthma in Grade 9** |  |  |  |  |  |  |
| No | 516,997 | 0.0 |  | 20,934 | 0.0 |  |
| Mild/moderate controlled | 15,170 | 6.0 | [5.2,6.8] | 787 | 8.2 | [3.5,12.9] |
| Severe controlled | 1,196 | 10.5 | [7.9,13.2] | 74 | 4.7 | [-13.7,23.1] |
| Mild/moderate uncontrolled | 1,464 | -10.1 | [-13.0,-7.1] | 141 | -7.7 | [-19.6,4.2] |
| Severe uncontrolled | 222 | -2.9 | [-9.9,4.0] | 9 | -3.2 | [-42.7,36.2] |
|  |  |  |  |  |  |  |
| **Non-eligibility to upper secondary school** | **n** | **OR** | **[95% CI]** | **n** | **OR** | **[95% CI]** |
| **Asthma in Grades 7-8** |  |  |  |  |  |  |
| No | 506,210 | 1.00 |  | 20,293 | 1.00 |  |
| Mild/moderate controlled | 25,442 | 0.82 | [0.77,0.86] | 1,410 | 0.78 | [0.69,0.88] |
| Severe controlled | 832 | 0.60 | [0.43,0.86] | 53 | 1.03 | [0.57,1.86] |
| Mild/moderate uncontrolled | 2,377 | 1.17 | [1.02,1.34] | 176 | 1.06 | [0.77,1.46] |
| Severe uncontrolled | 188 | 0.69 | [0.36,1.32] | 13 | 2.05 | [0.64,6.52] |
|  |  |  |  |  |  |  |
| **Asthma in Grade 9** |  |  |  |  |  |  |
| No | 516,997 | 1.00 |  | 20,934 | 1.00 |  |
| Mild/moderate controlled | 15,170 | 0.78 | [0.73,0.84] | 787 | 0.80 | [0.68,0.94] |
| Severe controlled | 1,196 | 0.78 | [0.60,1.01] | 74 | 1.02 | [0.61,1.71] |
| Mild/moderate uncontrolled | 1,464 | 1.49 | [1.26,1.76] | 141 | 1.21 | [0.85,1.72] |
| Severe uncontrolled | 222 | 0.93 | [0.55,1.56] | 9 | 0.91 | [0.22,3.77] |

^a^ Adjusted models are adjusted for gender, mother's and father's education and asthma, family income the year the child started school

## **Table A4.** Association between asthma and grade point sum/non-eligibility to upper secondary school by highest parental education

|  | **Middle school** | | | **Upper secondary school** | | | **College/university** | | |
| --- | --- | --- | --- | --- | --- | --- | --- | --- | --- |
|  | **Adjusted^a^** | | | **Adjusted^a^** | | | **Adjusted^a^** | | |
| **Grade point sum** | **n** | **β** | **[95% CI]** | **n** | **β** | **[95% CI]** | **n** | **β** | **[95% CI]** |
| Asthma in Grades 7-8 | 24,156 | 2.8 | [-1.1,6.6] | 288,083 | 5.0 | [4.1,5.9] | 244,755 | 2.8 | [1.9,3.6] |
| Asthma in Grade 9 | 24,156 | 2.8 | [-2.1,7.7] | 288,083 | 6.2 | [5.1,7.3] | 244,755 | 3.5 | [2.4,4.5] |
|  |  |  |  |  |  |  |  |  |  |
| **Non-eligibility to upper secondary school** | **n** | **OR** | **[95% CI]** | **n** | **OR** | **[95% CI]** | **n** | **OR** | **[95% CI]** |
| Asthma in Grades 7-8 | 24,156 | 0.97 | [0.85,1.11] | 288,083 | 0.82 | [0.78,0.87] | 244,755 | 0.77 | [0.69,0.86] |
| Asthma in Grade 9 | 24,156 | 0.98 | [0.82,1.16] | 288,083 | 0.83 | [0.77,0.89] | 244,755 | 0.80 | [0.71,0.92] |
|  |  |  |  |  |  |  |  |  |  |
| **Grade point sum** | **n** | **β** | **[95% CI]** | **n** | **β** | **[95% CI]** | **n** | **β** | **[95% CI]** |
| **Asthma in Grades 7-8** |  |  |  |  |  |  |  |  |  |
| No | 23,094 | 0.0 |  | 272,364 | 0.0 |  | 231,045 | 0.0 |  |
| Mild/moderate controlled | 911 | 5.6 | [1.6,9.7] | 13,747 | 5.7 | [4.8,6.6] | 12,194 | 3.5 | [2.6,4.4] |
| Severe controlled | 14 | 41.1 | [5.8,76.3] | 437 | 17.4 | [12.4,22.5] | 434 | 7.3 | [3.1,11.4] |
| Mild/moderate uncontrolled | 132 | -20.0 | [-31.2,-8.8] | 1,427 | -5.7 | [-8.8,-2.6] | 994 | -8.6 | [-11.9,-5.4] |
| Severe uncontrolled | 5 | -10.6 | [-56.5,35.2] | 108 | 2.2 | [-9.6,14.0] | 88 | 5.3 | [-4.5,15.1] |
| **Asthma in Grade 9** |  |  |  |  |  |  |  |  |  |
| No | 23,540 | 0.0 |  | 278,222 | 0.0 |  | 236,169 | 0.0 |  |
| Mild/moderate controlled | 500 | 8.3 | [3.3,13.3] | 8,211 | 7.8 | [6.6,9.0] | 7,246 | 4.0 | [2.9,5.1] |
| Severe controlled | 27 | 7.7 | [-19.5,34.9] | 605 | 15.0 | [10.8,19.2] | 638 | 7.4 | [3.6,11.2] |
| Mild/moderate uncontrolled | 81 | -32.3 | [-48.4,-16.2] | 918 | -12.3 | [-16.4,-8.2] | 606 | -6.3 | [-10.5,-2.1] |
| Severe uncontrolled | 8 | -2.2 | [-34.0,29.6] | 127 | -8.3 | [-18.3,1.7] | 96 | 2.4 | [-6.8,11.6] |
|  |  |  |  |  |  |  |  |  |  |
| **Non-eligibility to upper secondary school** | **n** | **OR** | **[95% CI]** | **n** | **OR** | **[95% CI]** | **n** | **OR** | **[95% CI]** |
| **Asthma in Grades 7-8** |  |  |  |  |  |  |  |  |  |
| No | 23,094 | 1.00 |  | 272,364 | 1.00 |  | 231,045 | 1.00 |  |
| Mild/moderate controlled | 911 | 0.93 | [0.80,1.07] | 13,747 | 0.80 | [0.75,0.84] | 12,194 | 0.75 | [0.67,0.84] |
| Severe controlled | 14 | 0.89 | [0.28,2.82] | 437 | 0.70 | [0.51,0.98] | 434 | 0.42 | [0.20,0.90] |
| Mild/moderate uncontrolled | 132 | 1.31 | [0.93,1.86] | 1,427 | 1.11 | [0.96,1.29] | 994 | 1.15 | [0.85,1.56] |
| Severe uncontrolled | 5 | 1.29 | [0.25,6.66] | 108 | 0.99 | [0.55,1.78] | 88 | 0.84 | [0.26,2.73] |
|  |  |  |  |  |  |  |  |  |  |
| **Asthma in Grade 9** |  |  |  |  |  |  |  |  |  |
| No | 23,540 | 1.00 |  | 278,222 | 1.00 |  | 236,169 | 1.00 |  |
| Mild/moderate controlled | 500 | 0.81 | [0.66,0.99] | 8,211 | 0.77 | [0.71,0.83] | 7,246 | 0.76 | [0.66,0.88] |
| Severe controlled | 27 | 1.36 | [0.63,2.95] | 605 | 0.67 | [0.50,0.89] | 638 | 1.00 | [0.66,1.52] |
| Mild/moderate uncontrolled | 81 | 2.31 | [1.51,3.54] | 918 | 1.48 | [1.25,1.76] | 606 | 1.01 | [0.67,1.52] |
| Severe uncontrolled | 8 | 0.72 | [0.15,3.50] | 127 | 1.05 | [0.61,1.81] | 96 | 0.96 | [0.30,3.05] |

^a^ Adjusted models are adjusted for gender, ADHD, gender x ADHD interaction and asthma, family income the year the child started school
